# Comparison of aneurysmal subarachnoid hemorrhage grading scores in patients with aneurysm clipping and coiling

**DOI:** 10.1038/s41598-020-66160-0

**Published:** 2020-06-08

**Authors:** Yuanjian Fang, Jianan Lu, Jingwei Zheng, Haijian Wu, Camila Araujo, Cesar Reis, Cameron Lenahan, Suijun Zhu, Sheng Chen, Jianmin Zhang

**Affiliations:** 1 Department of Neurosurgery, The Second Affiliated Hospital, School of Medicine, Zhejiang University, Hangzhou, Zhejiang, China; 2 Department of Physiology and Pharmacology, Loma Linda University School of Medicine, Loma Linda, CA USA; 3 Center for Neuroscience Research, Loma Linda University School of Medicine, Loma Linda, CA USA; 4 Burrell College of Osteopathic Medicine, Las Cruces, NM USA; 5 Department of Neurosurgery, First People’s Hospital of Yuhang District, Hangzhou, Zhejiang, China; 6 Brain Research Institute, Zhejiang University, Hangzhou, Zhejiang, China; 7 Collaborative Innovation Center for Brain Science, Zhejiang University, Hangzhou, Zhejiang, China

**Keywords:** Stroke, Stroke

## Abstract

Past studies revealed the prognosis differed between aneurysmal subarachnoid hemorrhage (aSAH) patients with surgical clipping and endovascular coiling. We retrospectively reviewed aSAH patients in our institution to investigate the effectiveness of grading scores between two groups. In the surgical clipping group (n = 349), VASOGRADE had a favorable performance for predicting delayed cerebral ischemia (DCI) (area under curve (AUC) > 0.750), and had better results than clinical (World Federation of Neurosurgical Societies (WFNS), Hunt & Hess (HH) and radiological scores (modified Fisher Scale (mFS), Subarachnoid Hemorrhage Early Brain Edema Score) (*P* < 0.05). Clinical and combined scores (VASOGRADE, HAIR) had favorable performance for predicting poor outcome (AUC > 0.750), and had better results than radiological scores (*P* < 0.05). In the coiling group (n = 320), none of the grading scores demonstrated favorable predictive accuracy for DCI (AUC < 0.750). Only WFNS and VASOGRADE had AUC > 0.700, with better performance than mFS (*P* < 0.05). The clinical and combined scores showed favorable performance for predicting a poor outcome (AUC > 0.750), and were better than the radiological scores (*P* < 0.05). Radiological scores appeared inferior to the clinical and combined scores in clipping and coiling groups. VASOGRADE can be an effective grading score in patients with clipping or coiling for predicting DCI and poor outcome.

## Introduction

Numerous prognostic grading systems have been proposed to predict clinical outcome, and to guide treatment and management after aneurysmal subarachnoid hemorrhage (aSAH)^1,2^. These prognostic grading scores can be divided into clinical, radiological, and combined scores. Clinical scores primarily focus on the patients’ mental status and neurological deficits that reflect the severity and changes in brain injury following aSAH. Hunt-Hess (HH) and World Federation of Neurological Society (WFNS) scales are the most commonly used clinical scores in the regular treatment and assessment of aSAH patients^3^. In contrast, the radiological scores assess the severity of the condition by quantifying imaging metrics, such as the amount and location of bleeding, as well as the degree of cerebral edema depicted on Computed Tomography (CT)^3^. As the most widely accepted radiological scores, the modified Fisher scale (mFS) quantifies the amount of bleeding to predict the incidence of vasospasm and delayed cerebral ischemia (DCI)^4^. Some recent radiological scores, such as the Subarachnoid Hemorrhage Early Brain Edema Score (SEBES)^5^ and the Global Cerebral Edema (GCE) score, predict the incidence of DCI or outcome by quantifying the degree of cerebral edema^6^. Because of the limitations and one-sidedness of the evaluation index, clinical or radiological scores may potentially underestimate the importance of each other. Consequently, some scores were developed to combine clinical and radiological factors for predicting outcome, such as VASOGRADE, HAIR and the SAH Score^7,8^. However, the accuracy and validity of those scores are debatable^1,9,10^. One study also pointed out the combined scores was shown to have no superiority when compared with the other single scores in aSAH patients^1^. Furthermore, the radiological scores had the poorest performance when predicting the outcome when compared with other scores^1,9^. These seem to deviate from the initial score results.

The surgical clipping and endovascular coiling groups had significantly different outcomes after aSAH. Coiling yielded a better clinical outcome in many large prospective randomized studies^11^. The effectiveness of grading scores may differ among aneurysm patients treated with clipping or coiling due to the different clinical courses. Herein, we compared the values of various grading systems in patients treated with clipping or coiling. The evaluation of different grading scores in different aSAH patients may provide neurologists with an optimal tool to predict outcomes.

## Methods

### Patients selection

With approval from the Institutional Review Board of Second Affiliated Hospital of Zhejiang University, this study retrospectively reviewed SAH patients admitted to our neurosurgery department between January 2014 and December 2015, with a confirmatory radiographic diagnosis or lumbar puncture included. Because the study was retrospective, the institutional review board determined that patient informed consent was not required. All methods were performed in accordance with relevant guidelines and regulations.

The inclusion criteria included patients with spontaneous SAH. Exclusion criteria included angiogram-negative patients, patients with history of trauma or previous brain injury (i.e. stroke, hemorrhage, surgery, etc., which left associated chronic changes on CT), arteriovenous malformation, missing radiological data, presence of serious comorbidities before SAH onset (i.e. coagulation defects, uncontrollable hypertension, arrhythmia, etc.) and initial radiological assessments performed more than 3 days after SAH onset. Those patients underwent external ventricular drainage (EVD) or decompressive craniotomy only, or conservative treatment were also excluded. It should be mentioned that intra-parenchymal hemorrhage was a common phenomenon of severe SAH, so those patients with diffused SAH with associated intra-parenchymal hemorrhage were also included in this study.

Routine CT scans were conducted on all patients at admission to evaluate the severity of SAH. The presence of an aneurysm was confirmed via digital subtraction angiography (DSA). Surgical and endovascular treatments were performed by neurosurgeons within 3 days following hospital admission. The decision to perform surgical clipping or endovascular coiling was determined by aneurysm-related factors (i.e. geometry and location). The aSAH patients were divided into clipping and coiling groups, and treated according to available guidelines^12^.

### Baseline characteristics and scores

For patients’ characteristics, the following data were recorded: age, sex, history of drinking or smoking, hypertension, hyperlipidemia, diabetes, aneurysm location (anterior circulation, posterior circulation or multiple aneurysms), aneurysm sizes, SAH-related complications (DCI, hydrocephalus (defined as radiological ventricular enlargement or clinical symptoms appeared^13^), rebleeding (defined as new or expanded hemorrhage^8^), and presence of seizure. Information pertaining to size and location of aneurysm were collected from angiographic records.

The clinical scores, including HH^14^ and WFNS^15^, and radiological scores, such as mFS^4^ and SEBES^5^, were reviewed. Combined scores including VASOGRADE, HAIR, and the SAH Score were evaluated according to the original criteria of each score^8,16,17^. HH and WFNS were reviewed from the medical history that were documented at admission. MFS and SEBES were independently scored by two blinded neurosurgeons. An independent third examiner was used when there was a discrepancy between the two blinded neurosurgeons.

### Outcome measures

The presence of DCI during hospitalization was used in this analysis as the primary outcome measure. The definition of DCI followed the criteria of previous studies^18,19^. Briefly, DCI was defined as clinical cerebral vasospasm (clinical deterioration that excluded other causes), or cerebral infarction (new cerebral infarction appeared on CT or MRI, which should exclude infarctions that appear within 48 hours after surgery or coiling).

The second outcome measure was the development of poor outcome, defined as a modified Rankin Scale (mRS)^20^ ranging from 3 to 6, and assessed at 3 months after discharge. An additional dichotomization of mRS (poor outcome defined as mRS 4 to 6) was also used in the analysis of the supplemental data. The data of mRS were obtained using telephone follow-up or outpatient follow-up records.

### Statistical analysis

Statistical analysis was performed using SPSS 22.0 (SPSS Institute, Chicago, IL, USA) and MedCalc Statistical Software version 18.2.1 (MedCalc Software bvba, Ostend, Belgium; http://www.medcalc.org; 2018). Continuous variables are expressed as the mean with standard error (SD). Categorical variables are expressed as frequency and percentage. Comparisons between groups were performed using the parametric t-test for continuous parameters and the Chi-square test or Fisher’s exact test for categorical parameters. *P* < 0.05 was considered statistically significant.

To assess the grading scores’ predictive performance for DCI and poor outcome in different groups after aSAH, the trends between grade and either DCI or poor outcome rate were analyzed using the cochairman-Armitage trend test^21,22^. Binary logistic regression analysis was performed with grading scores to evaluate the odds ratio (OR) and 95% confidence interval (CI)^1^. Receiver operating characteristic (ROC) curves were calculated to evaluate the area under the ROC curve (AUC), estimating the discrimination of grading scores^23^. An AUC greater than 0.750 was considered a good predictive accuracy (discrimination) of scores^24–26^. AUCs were compared using Delong test, and *P* < 0.05 was considered statistically significant^27^.

## Results

### Baseline characteristics

**Figure 1:**
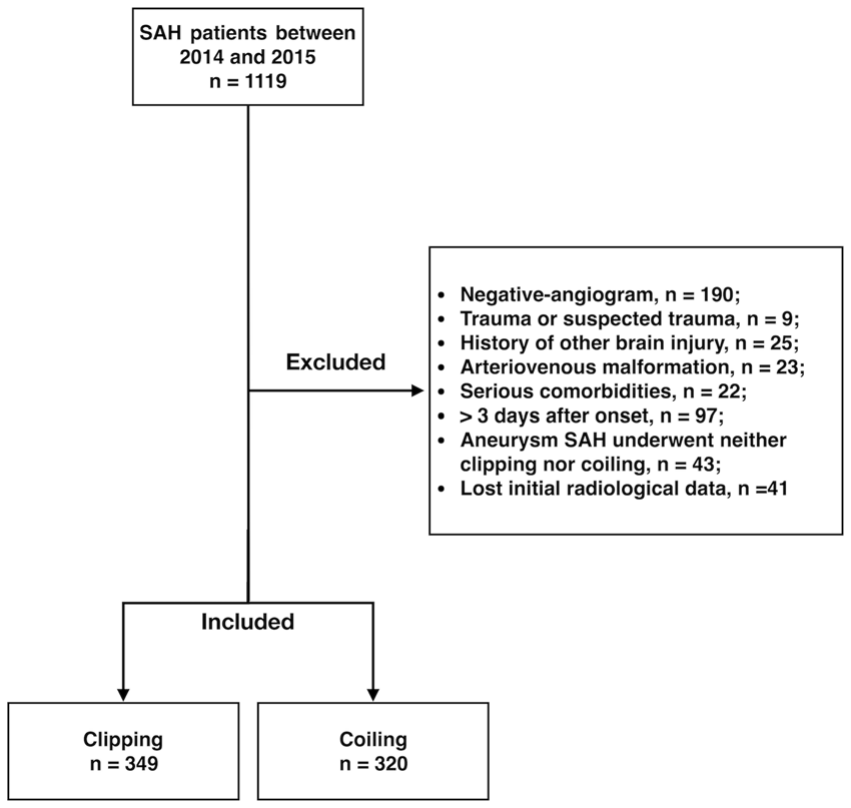
Flowchart of subarachnoid hemorrhage (SAH) patients.

From January 2014 to December 2015, a total of 1,119 patients with SAH were admitted to our hospital. The following patients were excluded before analysis: 190 patients with negative angiogram, 9 patients with history of trauma or suspected trauma, 25 patients with a history of other brain injuries, 23 patients diagnosed with arteriovenous malformation, 22 patients accompanied with serious comorbidities, 97 patients who arrived at the hospital more than three days after onset of symptoms, 43 patients with aneurysm who neither underwent clipping nor coiling, and 41 patients with missing radiological data. In total, 669 aSAH patients were included in our analysis. In 669 aneurysmal patients, 349 patients underwent clipping and 320 patients underwent coiling (Fig. 1).

The baseline characteristics between clipping patients and coiling patients had a randomized effect in age, sex, drinking, hypertension, hyperlipidemia, diabetes, and clinical and radiological data at admission, including WFNS, HH, mFS, and SEBES. However, clipping patients had a higher proportion of smokers (27.2% vs 19.4% in coiling, *P* = 0.017), anterior circulation aneurysm (66.5% vs 53.8% in coiling, *P* < 0.001), and larger aneurysms (4.7 ± 2.0 mm vs 4.2 ± 2.4 mm in coiling, *P* = 0.005). Clipping patients had a longer length of hospital stay (LOS) compared with coiling patients (15.6 ± 11.3 days vs 12.7 ± 8.2 days, *P* < 0.001). Patients that underwent clipping had a DCI rate more than double that of patients that underwent coiling (38.1% vs 15.9%, *P* < 0.001). The poor outcome rate was higher in clipping patients (54.7% vs 35.9% in coiling, *P* < 0.001) (Table 1).

**Table 1:** Characteristics of aneurysmal SAH patients between 2014 and 2015.

		All patients (n = 669)	Clipping (n = 349)	Coiling (n = 320)	*P* Value
Age (year, mean ± SD)		55.9 ± 11.1	56.3 ± 10.1	55.5 ± 12.1	0.333
Sex (male, %)		248 (37.1)	136 (39.0)	112 (35.0)	0.288
Smoking (%)		157 (23.5)	95 (27.2)	62 (19.4)	0.017
Drinking (%)		104 (15.5)	60 (17.2)	44 (13.8)	0.083
Hypertension (%)		255 (38.1)	139 (39.8)	116 (36.3)	0.341
Hyperlipidemia (%)		235 (35.1)	124 (35.5)	111 (34.7)	0.820
Diabetes (%)		25 (3.7)	13 (3.7)	12 (3.8)	0.986
WFNS (%)	1	441 (65.9)	231 (66.2)	210 (65.6)	0.997
	2	38 (5.7)	20 (5.7)	18 (5.6)	
	3	43 (6.4)	23 (6.6)	20 (6.3)	
	4	99 (14.8)	50 (14.3)	49 (15.3)	
	5	48 (7.2)	25 (7.2)	23 (7.2)	
HH (%)	1	72 (10.8)	31 (8.9)	41 (12.8)	0.323
	2	373 (55.8)	204 (58.5)	169 (52.8)	
	3	111 (16.6)	60 (17.2)	51 (15.9)	
	4	94 (14.1)	46 (13.2)	48 (15.0)	
	5	19 (2.8)	8 (2.3)	11 (3.4)	
mFS (%)	0	28 (4.2)	12 (3.4)	16 (5.0)	0.283
	1	70 (10.5)	32 (9.2)	38 (11.9)	
	2	146 (21.8)	77 (22.1)	69 (21.6)	
	3	155 (23.2)	91(26.1)	64 (20.0)	
	4	270 (40.4)	137 (39.3)	133 (41.6)	
SEBES (%)	0	154 (23.0)	79 (22.6)	75 (23.4)	0.608
	1	81 (12.1)	41 (11.7)	40 (12.5)	
	2	103 (15.4)	53 (15.2)	50 (15.6)	
	3	70 (10.5)	43 (12.3)	27 (8.4)	
	4	261 (39.0)	133 (38.1)	128 (40.0)	
Aneurysm location (%)					**<0.001**
	Anterior circulation	404 (60.4)	232 (66.5)	172 (53.8)	
	Posterior circulation	223 (33.3)	89 (25.5)	134 (41.9)	
	Multiple aneurysms	42 (60.9)	28 (8.0)	14 (4.4)	
Aneurysm sizes (mm, mean ± SD)^‡^		4.4 ± 2.2	4.2 ± 2.4	4.7 ± 2.0	**0.005**
LOS (day, mean ± SD)		14.2 ± 10.0	15.6 ± 11.3	12.7 ± 8.2	**<0.001**
Complications (%)					
	DCI	184 (27.5)	133 (38.1)	51 (15.9)	<**0.001**
	Hydrocephalus	100 (14.9))	55 (15.8)	45 (14.1)	0.539
	Rebleeding	13 (1.9))	6 (1.7)	7 (2.2)	0.661
	Seizure	14 (2.1)	8 (2.3)	6 (1.9)	0.706
Outcome (mRS, %)	0-2	363 (54.3)	158 (45.3)	205 (64.1)	**<0.001**
	3-6	234 (45.7)	191 (54.7)	115 (35.9)	

^‡^Counted in 579 single aneurysmal patients, 38 patients lost data of aneurysm size in clipping group, 10 patients lost data of aneurysm size in the coiling group.

### Predictive performance of grading scores in clipping group

**Figure 2:**
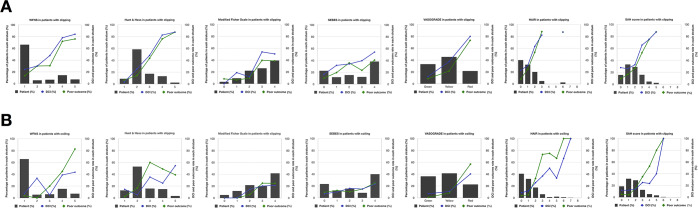
Distributions and corresponding delayed cerebral ischemia (DCI) rate and poor outcome rate in each score. (**A**) Clipping group; (**B**) Coiling group.

There was no patient distribution in HAIR grades 4, 5, 7, or 8, nor was there patient distribution in The SAH Score of grades 6, 7, or 8 (Fig. 2A). In predicting DCI, each presented a strong trend between increased score and DCI rate (*P* for trend <0.001) (Fig. 2A). Each score predicted DCI with a good correlation. VASOGRADE (OR = 5.421) had the highest OR value, followed by HH, HAIR, WFNS, mFS, the SAH Score, and SEBES (both OR > 1) (Table 2).

**Table 2:** OR and AUC of each score for predicting DCI and poor outcome.

	Clipping				Coiling			
	OR	95%CI	AUC*	95%CI	OR	95%CI	AUC*	95%CI
**For predicting DCI**								
WFNS	2.077	1.729-2.495	0.720	0.670–0.767	1.811	1.480–2.216	0.735	0.683–0.783
HH	2.875	2.143–3.858	0.709	0.658–0.756	1.973	1.478–2.634	0.692	0.639–0.742
mFS	2.051	1.613–2.608	0.682	0.630–0.730	1.674	1.232–2.277	0.634	0.579–0.687
SEBES	1.505	1.296–1.749	0.670	0.618–0.719	1.406	1.141–1.733	0.640	0.585–0.639
VASOGRADE	5.421	3.632–8.091	**0.771**	0.724–0.814	3.432	2.175–5.417	0.725	0.673–0.773
HAIR	2.259	1.748–2.919	0.708	0.657–0.755	1.706	1.369–2.126	0.692	0.638–0.742
The SAH Score	1.811	1.473–2.226	0.671	0.619–0.720	1.416	1.131–1.773	0.612	0.556–0.666
**For predicting poor outcome**								
WFNS	2.289	1.898–2.762	**0.785**	0.738–0.827	3.135	2.395–4.105	**0.865**	0.823–0.901
HH	4.103	2.946–5.714	**0.773**	0.725–0.815	3.832	2.695–5.448	**0.818**	0.771–0.858
mFS	1.983	1.521–2.585	0.671	0.619–0.720	2.246	1.565–3.224	0.692	0.638–0.742
SEBES	1.458	1.238–1.717	0.654	0.602–0.704	1.365	1.115–1.607	0.632	0.576–0.685
VASOGRADE	6.123	3.962–9.474	**0.786**	0.739–0.827	13.802	7.071–26.940	**0.869**	0.827–0.904
HAIR	3.423	2.495–4.697	**0.797**	0.751–0.835	3.582	2.520–5.091	**0.855**	0.811–0.891
The SAH Score	2.352	1.848–2.993	0.740	0.691–0.785	2.691	2.029–3.570	**0.802**	0.754–0.844

*AUC > 0.750 was considered as favorable predictive accuracy.

**Figure 3:**
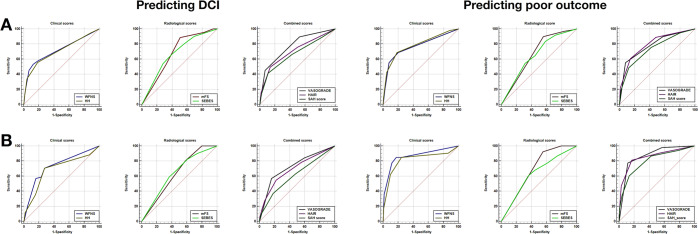
Receiver operating characteristic (ROC) of clinical, radiological and combined scores in clipping group and coiling group. (**A**) Clipping group; (**B**) Coiling group.

Only VASOGRADE (AUC = 0.771) showed favorable predictive accuracy for DCI. The AUC values of WFNS, HH, HAIR, mFS, SEBES, and the SAH Score were under 0.750 (Table 2) (Fig. 3A). In all scores, VASOGRADE showed a better predictive accuracy of DCI, whether compared to clinical scores (*P* = 0.009, vs WFNS; *P* = 0.003, vs HH), radiological scores (*P* < 0.001), or combined scores (*P* = 0.019, vs HAIR; *P* < 0.001, vs The SAH Score) **(**Table 3**)**.

**Table 3:** The AUC compared between clinical, radiological and combined scores.

	Clipping		Coiling	
	*P* value of predicting DCI	*P* value of predicting poor outcome	*P* value of predicting DCI	*P* value of predicting poor outcome
**Compared between clinical and radiological scores**				
WFNS vs mFS	0.267	**0.006**	**0.027**	**<0.001**
WFNS vs SEBES	0.141	**0.002**	0.073	**<0.002**
HH vs mFS	0.437	**0.002**	0.225	**<0.003**
HH vs SEBES	0.259	**<0.001**	0.346	**0.005**
**Compared between combined and single scores**				
VASOGRADE vs WFNS	**0.009**	0.547	0.732	0.823
VASOGRADE vs HH	**0.003**	0.973	0.352	0.078
VASOGRADE vs mFS	**<0.001**	**<0.001**	**0.009**	**<0.001**
VASOGRADE vs SEBES	**<0.001**	**<0.001**	0.098	**<0.001**
HAIR vs WFNS	0.648	0.330	0.317	0.744
HAIR vs HH	0.960	0.604	0.992	0.280
HAIR vs mFS	0.473	**<0.001**	0.254	**<0.001**
HAIR vs SEBES	0.346	**<0.001**	0.402	**<0.001**
The SAH Score vs WFNS	0.050	0.143	**<0.001**	**<0.021**
The SAH Score vs HH	0.136	**0.042**	**0.020**	**0.628**
The SAH Score vs mFS	0.780	0.080	0.645	**0.006**
The SAH Score vs SEBES	0.984	**0.040**	0.657	**0.004**

For predicting poor outcome, each score showed a good trend between increased scores and poor outcome rate (*P* for trend <0.001) (Fig. 2A). The OR values were considerably correlated with poor outcome. The VASOGRADE (OR = 6.123) had the highest OR value, followed by HH, HAIR, The SAH Score, WFNS, mFS, and SEBES (both OR > 1) (Table 2).

The clinical scores, WFNS (AUC = 0.785) and HH (AUC = 0.773), and the two combined scores, VASOGRADE (AUC = 0.786) and HAIR (AUC = 0.797), showed favorable predictive accuracy for poor outcome (Table 2) (Fig. 3A). The performance of radiological scores was poorer when compared with the clinical and combined scores. The AUC of mFS (AUC = 0.671) was significantly lower than WFNS, HH, VASOGRADE, and HAIR (both *P* ≤ 0.006). Similarly, the AUC of SEBES (AUC = 0.654) was significantly lower than that of WFNS, HH, VASOGRADE, and the SAH Score (both *P* ≤ 0.040). It should be mentioned that the AUC of the SAH Score was significantly higher than the AUC of SEBES (P = 0.040), but lower than HH (P = 0.042) (Table 3).

The ability of each score to predict mRS ranging from 3 to 6 were consistent with the ability to predict mRS ranging from 4 to 6 (Supplemental Table 1).

### Predictive performance of grading scores in coiling group

Distributions of each score are shown in Fig. 2B, HAIR had no patients distributed in grade 8, and The SAH Score had no patients distributed in grades 7 or 8. For predicting DCI, clinical scores (both *P* for trend <0.001), radiological scores (both *P* for trend = 0.001), and combined scores (VASOGRADE and HAIR, *P* for trend <0.001 and the SAH Score, *P* for trend = 0.002), presented a good trend between increased score and DCI rate. Furthermore, both were significantly associated with an increased incidence of DCI, with the VASOGRADE scores demonstrating the strongest association (OR = 3.432), followed by the HH, WFNS, HAIR, mFS, the SAH Score (both OR > 1) (Table 2).

None of the grading scores showed sufficient discrimination (AUC > 0.750). Only WFNS (AUC = 0.735) and VASOGRADE (AUC = 0.725) were over AUC 0.700. (Table 2) (Fig. 3B). WFNS had the most accurate predictive performance among clinical, radiological, and combined scores, particularly when compared with mFS (*P* = 0.027) and The SAH Score (*P* < 0.001). Besides, the AUC of VASOGRADE was the second highest, and significantly increased compared to mFS (*P* = 0.009). The AUC of The SAH Score was the lowest, and significantly lower than WFNS (*P* < 0.001) and HH (*P* = 0.020) (Table 3).

For predicting poor outcomes, all grading scores, including WFNS, HH, mFS, VASOGRADE, HAIR, the SAH Score (both *P* for trend <0.001), and SEBES (*P* for trend = 0.003), presented a good trend (Fig. 2B). Similar to its capability in predicting DCI, VASOGRADE (OR = 13.802) showed the strongest statistical association with poor outcome, followed by HH, HAIR, WFNS, The SAH Score, mFS, and SEBES (both OR > 1) (Table 2).

Clinical scores, including WFNS (AUC = 0.865), HH (AUC = 0.818), and combined scores, such as VASOGRADE (AUC = 0.869), HAIR (AUC = 0.855), and The SAH Score (AUC = 0.802), showed favorable predictive accuracy. However, radiological scores, mFS (AUC = 0.692), and SEBES (AUC = 0.632) are not satisfactory in comparing AUC (Table 2) (Fig. 3B). The predictive accuracy of radiological scores was significantly lower than clinical scores (vs mFS, both *P* ≤ 0.003; vs SEBES, both *P* ≤ 0.005) and combined scores (vs mFS, both *P* < 0.001 (except The SAH Score); vs SEBES, both *P* < 0.001). The AUC of the SAH Score was significantly lower than WFNS (*P* = 0.021)

The performances of each score to predict mRS between 3 and 6 were consistent with the performance in predicting mRS between 4 and 6 (Supplemental Table 1).

## Discussion

### Choice of grading scores in patients with clipping and coiling

In this study, we retrospectively compared the performance of different grading scores for predicting DCI and poor outcome in patients with surgery clipping and endovascular coiling. The performance of different grading scores varied in each patient group. However, we found that VASOGRADE maintained a leading predictive accuracy, whether in clipping or coiling patients. The radiological scores showed poor predictive power in each group of patients. The predictive performances of clinical and combined scores were acceptable and comparable between the two groups for predicting poor outcome (except The SAH Score in clipping group), but varied for predicting DCI.

In clipping patients, VASOGRADE may be the first choice for predicting DCI. The clinical scores and the other combined scores had a similar power to predict DCI, which was significantly better than the radiological scores (*P* < 0.05). Clinical scores, such as WFNS and HH, as well as combined scores, such as HAIR and VASOGRADE, can be optimal in predicting poor outcomes. It should be mentioned that the SAH Score was neither accurate nor superior compared to the HH in this study (*P* = 0.042).

In coiling patients, WFNS and VASOGRADE were recommended to predict DCI, despite no scores showing favorable predictive accuracy, and the statistical significance was only shown when compared to the mFS (*P* < 0.05). Additionally, the performance of the SAH Score was significantly lower than the clinical scores, which seems contrary to its intended purpose. Both clinical and combined scores showed favorable performance for predicting poor outcome, and were also significantly better than the radiological scores (*P* < 0.05). Similarly, when predicting DCI, the performance of the SAH Score was significantly lower than WFNS (*P* = 0.021).

### Performance of grading scores in literature

The predictive performance of clinical, radiological, and combined scores have been investigated previously. However, the results of comparison were conflicting in various studies^1,5,9,10,17,28^. In reviewing the literature, a recent study, based on 423 aSAH patients, compared three types of scores, and found that the combined grading scores (VASOGRADE, HAIR) have no superiority to clinical scores (WFNS, HH), whether in predicting cerebral infarction or unfavorable outcome. Additionally, the radiological scores (mFS, Barrow Neurological Institute Grading Scale (BNI)^29^) had the poorest predictive performance in scores of three categories^1^. Another study comparing grading scores had 279 aSAH patients, and the radiological scores had poor predictive performance. The AUCs of BNI (AUC 0.684 and 0.680 for predicting unfavorable outcome and mortality) and mFS (AUC 0.604 and 0.554 for predicting unfavorable outcome and mortality) at discharge were significantly lower than HH (AUC 0.806 and 0.782 for predicting unfavorable outcome and mortality) and WFNS (AUC 0.785 and 0.740 for predicting unfavorable outcome and mortality)^9^. In the study regarding the SEBES score, mFS presented with the poorest AUC value (AUC = 0.66) for predicting unfavorable outcome (mRS score of 4 to 6 at 3 months) when compared with clinical scores (WFNS, HH). However, no difference was found in predicting DCI, although there was no AUC higher than 0.750 (AUC = 0.60 for WFNS, AUC = 0.56, for HH, AUC = 0.58 for mFS)^5^. However, the predictive performance of SEBES in our study was not as desirable as described in the initial study. While in another study, it was found that the combined score, HAIR, had an increased AUC value compared to the clinical score, HH^10^. Additionally, the initial study of The SAH Score showed a favorable AUC value of HH (AUC = 0.771) and WFNS (AUC = 0.777)^17^.

### Potential causes of performance discrepancy

The inconsistent results between the literature may be derived from the different patient cohorts consisting of different numbers of clipping, coiling, negative angiograms, and other treatments (i.e. EVD, decompressive craniotomy, or conservative treatment). The clipping patients varied from 37.2–59.1%, and coiling patients varied from 19.6–62.8% in the previous studies^1,5,9,10,17,28^. Generally, the aneurysms of clipping patients are more likely to be wide-necked, larger in size, and located in the anterior circulation^30^. Coiling presented with more benefit to the short-term outcome due the reduced invasiveness relative to clipping^11^. In this study, we confirmed these differences of characteristics, clinical course, and outcome in the clipping and coiling groups (Table 1). Thus, we thought that the cohorts with more coiling patients may have a relatively better outcome than the cohorts with more patients from the clipping group. The different outcome distributions may also reflect different results of the performance analysis of the grading scores. Therefore, we analyzed the performance of grading scores in patients that had underwent coiling or clipping.

Regarding the variation of clinical course and outcome in clipping and coiling patients, we observed that the performance of radiological scores was generally poor in both clipping and coiling patients, similar to the results of previous studies^1,5^. We speculate that this may due to differences regarding the evaluation index of the imaging score and the prognosis score. As we know, the prognostic assessment is only quantified by clinical symptoms (clinical cerebral vasospasm (a part of DCI), mRS, and Glasgow Outcome Scale, which was quantified from the ability of work and life change than radiological change). However, the radiological data may partially indicate the severity after SAH, the clinical symptoms of each patient with the same degree of bleeding or brain edema vary due to each individual’s unique tolerance.

VASOGRADE simply combined the data from clinical and radiological scores to predict DCI and poor outcome, and avoided inaccurate predictions for those with mild clinical symptoms and significant amount of bleeding^7^. A lower VASOGRADE score compared to other combined scores can improve the discrimination of VASOGRADE. Besides, the HAIR and The SAH Score had more sophisticated categorization, and were usually lost patients’ distribution in some grades which appears to have some problems in the patient distributions. In contrast to VASOGRADE, HAIR and the SAH Score were derived from multiple logistic regression models, therefore the accuracy is greatly limited by the prior factors included in the analysis. The characteristics and size of the patient cohort, especially the small sample size, impacted the performance of the predictive model. Meanwhile, these scores were initially invented to predict the in-hospital mortality rather than the DCI or poor outcome^8,17^. It showed no superiority to single grading systems, but their predictive performance was acceptable for predicting DCI and poor outcome.

## Limitations

Our study presented some limitations that should be addressed. First, the retrospective nature and single-center observational study design may introduce some potential biases. To limit this impact, all clinical scores were reviewed from the medical records at admission and reconfirmed by signs and symptoms documented at admission. Radiological scores and DCI confirmation were conducted by two examiners that were blinded to clinical information. There is a possibility that clinically silent infarctions were missed in our study, especially for those patients with mild symptoms and a short hospital stay. However, all patients in this study had a routine CT evaluation prior to being discharged from the hospital to avoid missing any clinically silent infarctions. The DCI rate was consistent with other studies (ranging from 21.0% to 31.3%)^5,29,31,32^. Second, the difference of dichotomization regarding mRS and the time of outcome evaluation may introduce some biases to the performance of different grading scores. We adopted the dichotomization of mRS (mRS scores of 1–2 defined as favorable outcome, and scores of 3–6 defined as poor outcome) and the assessment of outcome at 3 months after discharge, as utilized by previous studies^1,29^. We analyzed the predictive accuracy of each score by setting poor outcome with a definitive score of mRS > 3 (Supplemental Table 1), and the results were consistent with the former dichotomization. Future studies should validate the predictive performance for long-term prognosis. Third, differences in selection criteria for clipping versus coiling may introduce biases to future studies. There were more smokers in the clipping group than the coiling group. This potentially limits the generalizability of our results.

## Conclusion

The VASOGRADE may be the preferred choice in predicting DCI and poor prognosis in both patients with clipping and coiling. However, the radiological scores may be an auxiliary diagnosis to assess the severity of aSAH and timing of surgery. Herein, an effective selection of the appropriate grading scores will further guide and improve clinical treatment.

## Supplementary information

Supplemental Table 1.

## Data Availability

The datasets generated and analyzed during the current study are available from the corresponding author on reasonable request.
