# Supplementary material for: Comparison of aneurysmal subarachnoid hemorrhage grading scores in patients with aneurysm clipping and coiling
Source: Sci Rep. 2020 Jun 8;10:9199. doi: 10.1038/s41598-020-66160-0 (PMC7280262; doi:10.1038/s41598-020-66160-0)
Supplement: Supplementary file 1 — Supplemental Table 1. [file 41598_2020_66160_MOESM1_ESM.docx]

**Title:** Comparison of aneurysmal subarachnoid hemorrhage grading scores in patients with aneurysm clipping and coiling.

**Authors**: Yuanjian Fang ^1^, M.D*; Jianan Lu ^1^, MD*; Jingwei Zheng ^1^ M.D; Haijian Wu ^1^ M.D; Camila Araujo ^2^, M.D; Cesar Reis ^2^, M.D; Cameron Lenahan ^6,7^ B.S, Suijun Zhu ^3^, M.D; Sheng Chen ^1^, M.D and Jianmin Zhang ^1, 4, 5^ M.D & Ph.D

^1^ Department of Neurosurgery, The Second Affiliated Hospital, School of Medicine, Zhejiang University, Hangzhou, Zhejiang, China.

^2^ Department of Physiology and Pharmacology, Loma Linda University School of Medicine, Loma Linda, CA, USA

^3^ Department of Neurosurgery, First People's Hospital of Yuhang District, Hangzhou, Zhejiang, China.

^4^ Brain Research Institute, Zhejiang University, Hangzhou, Zhejiang, China.

^5^ Collaborative Innovation Center for Brain Science, Zhejiang University, Hangzhou, Zhejiang, China.

^6^ Center for Neuroscience Research, Loma Linda University School of Medicine, Loma Linda, CA, USA

^7^ Burrell College of Osteopathic Medicine, Las Cruces, NM, USA

*These authors contributed equally to this study.

**Correspondence**:

Sheng Chen, M.D and Jianmin Zhang, M.D&Ph.D; Department of Neurosurgery, The Second Affiliated Hospital, School of Medicine, Zhejiang University, NO.88 Jiefang Rd, 310009, Hangzhou, PR China. E-mail: saintchan@zju.edu.cn and zjm135@zju.edu.cn. TEL: +86-571-87784815; FAX: +86-571-87784755

| **Supplemental Table 1. OR and AUC of each score for predicting poor outcome (mRS 4-6)** | | | | | | | | | | | | | |
| --- | --- | --- | --- | --- | --- | --- | --- | --- | --- | --- | --- | --- | --- |
|  | **Clipping** | | | | |  | | **Coiling** | | | | |  |
|  | **OR** | **95%CI** | **AUC** | **95%CI** |  | | **OR** | | **95%CI** | **AUC** | **95%CI** |  |  |
| **WFNS** | 2.464 | 2.012-3.017 | **0.810** | 0.765-0.850 |  | | 3.907 | | 2.723-5.605 | **0.901** | 0.863-0.932 |  |  |
| **HH** | 4.280 | 3.034-6.039 | **0.815** | 0.770-0.854 |  | | 5.433 | | 3.491-8.456 | **0.883** | 0.843-0.916 |  |  |
| **mFS** | 1.999 | 1.477-2.701 | 0.668 | 0.616-0.717 |  | | 2.436 | | 1.595-3.721 | 0.702 | 0.649-0.752 |  |  |
| **SEBES** | 1.433 | 1.194-1.719 | 0.650 | 0.598-0.700 |  | | 1.355 | | 1.086-1.689 | 0.627 | 0.572-0.680 |  |  |
| **VASOGRADE** | 8.646 | 5.163-4.480 | **0.821** | 0.777-0.860 |  | | 30.394 | | 11.520-80.188 | **0.905** | 0.867-0.935 |  |  |
| **HAIR** | 3.524 | 2.501-4.965 | **0.835** | 0.792-0.872 |  | | 4.273 | | 2.834-6.443 | **0.897** | 0.858-0.928 |  |  |
| **SAH score** | 2.689 | 2.049-3.530 | **0.776** | 0.729-0.819 |  | | 3.482 | | 2.471-4.907 | **0.870** | 0.828-0.905 |  |  |
| Abbrevations: AUC, area under curve; CI, confidence interval; HH, Hunt Hess; mFS, modified Fisher scale; mRS, modified Rankin scale; OR, odd ratio; SAH, subarachnoid hemorrhage; SD, standard error; SEBES, Subarachnoid hemorrhage Early Brain Edema Score; WFNS, World Federation of Neurosurgical Societies. | | | | | | | | | | | | | |
